# Conversion of methylmercury into inorganic mercury via organomercurial lyase (MerB) activates autophagy and aggresome formation

**DOI:** 10.1038/s41598-023-47110-y

**Published:** 2023-11-15

**Authors:** Yasukazu Takanezawa, Kouhei Ishikawa, Shunsuke Nakayama, Ryosuke Nakamura, Yuka Ohshiro, Shimpei Uraguchi, Masako Kiyono

**Affiliations:** Department of Public Health, School of Pharmacy, Kitasato University, 5-9-1 Shirokane, Minato-ku, Tokyo, 108-8641 Japan

**Keywords:** Autophagy, Cell death, Molecular biology

## Abstract

Methylmercury (MeHg) is converted to inorganic mercury (iHg) in several organs; however, its impact on tissues and cells remains poorly understood. Previously, we established a bacterial organomercury lyase (MerB)-expressing mammalian cell line to overcome the low cell permeability of iHg and investigate its effects. Here, we elucidated the cytotoxic effects of the resultant iHg on autophagy and deciphered their relationship. Treatment of MerB-expressing cells with MeHg significantly increases the mRNA and protein levels of LC3B and p62, which are involved in autophagosome formation and substrate recognition, respectively. Autophagic flux assays using the autophagy inhibitor chloroquine (CQ) revealed that MeHg treatment activates autophagy in MerB-expressing cells but not in wild-type cells. Additionally, MeHg treatment induces the accumulation of ubiquitinated proteins and p62, specifically in MerB-expressing cells. Confocal microscopy revealed that large ubiquitinated protein aggregates (aggresomes) associated with p62 are formed transiently in the perinuclear region of MerB-expressing cells upon MeHg exposure. Meanwhile, inhibition of autophagic flux decreases the MeHg-induced cell viability of MerB-expressing cells. Overall, our results imply that cells regulate aggresome formation and autophagy activation by activating LC3B and p62 to prevent cytotoxicity caused by iHg. These findings provide insights into the role of autophagy against iHg-mediated toxicity.

## Introduction

Mercury is a harmful heavy metal that is widely distributed in nature. Humans and wildlife are mainly exposed to its organic form, methylmercury (CH_3_Hg^+^, MeHg), through the consumption of contaminated fish and seafood^1^. MeHg disrupts the development and functions of several organs, notably targeting the nervous system, and is thought to be a risk factor for many diseases, including diabetes and neurological diseases^2,3^. MeHg binds to thiol groups in biomolecules and forms various MeHg–substituted thiol complexes. Cysteine is considered the primary target of MeHg and is associated with the disruption of cellular functions. In particular, MeHg-mediated thiol modification affects the activity of protein kinase B (Akt)/cyclic adenosine monophosphate response element-binding protein (CREB) and kelch-like ECH-associated protein 1 (Keap1)/nuclear factor-erythroid 2-related factor 2 (Nrf2) signaling pathways^4,5^. Sulfur- and selenium-based amino acids are also targets of MeHg. For example, MeHg facilitates the formation of dehydroalanine in selenoenzymes^6^, which irreversibly impairs enzymatic function. Additionally, there has been a surge in evidence that MeHg induces epigenetic modifications, which may affect MeHg toxicity. However, currently, we have limited knowledge about the link between MeHg-induced epigenetic modifications and children’s neurodevelopment^7,8^.

Multiple studies have reported that MeHg is converted to inorganic mercury (Hg^2+^, iHg) via MeHg demethylation after incorporation into human and animal bodies. iHg is poorly absorbed by the gastrointestinal tract; thus, MeHg demethylation in the gut lumen via microbiota is proposed to decrease MeHg bioavailability in the body^9,10^. However, MeHg is demethylated in the liver and brain of animals^11,12^. Although the mechanism of MeHg demethylation is not well understood, the involvement of selenium (Se) in this process has been postulated^13^. This element contributes to HgSe precipitation and MeHg demethylation in aquatic organisms^14^. Converted iHg appears to persist in the tissues for months or years and continues to be the source of mercury exposure. Additionally, iHg can be detected in an ex vivo liver rat model^15^ and in vitro^16^ studies. Therefore, research on the effect of iHg on cells and tissues is particularly important in understanding the consequences of long-term MeHg toxicity.

**Figure 1:**
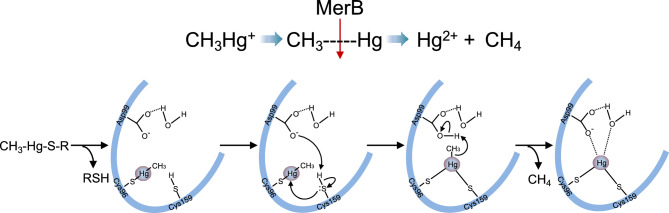
Scheme depicting the catalyzed reaction of MerB.

Although some studies have demonstrated the differences in toxicity and cellular effects of MeHg and iHg, accurate understanding has been limited owing to the decreased cellular permeability of iHg compared to MeHg. Furthermore, it is difficult to eliminate the effects of excess extracellular iHg in experimental systems, where the culture media are supplemented with iHg. Additionally, the mechanism of MeHg demethylation in mammals remains elusive. To address these concerns and overcome low cell permeability of iHg, we recently established a cell line (HEK293 cells) that utilizes the bacterial organomercurial lyase MerB^17^—the only known enzyme that demethylates MeHg and converts it to iHg—to investigate the effect of iHg. MerB is part of the *mer* operon, an elaborate mercury tolerance system in bacteria^18^. MerB catalyzes the breaking of carbon–mercury bonds through protonolysis of MeHg^19^ (Fig. 1). Established MerB-expressing mammalian cells produce iHg from the incorporated MeHg and elicit higher cytotoxic effects of iHg than those of MeHg^17^. Moreover, increased MeHg cytotoxicity is associated with MerB demethylation activity in a bacterial expression system^20^. These results suggest that demethylation of MeHg in situ may increase the toxicity of MeHg exposure.

Autophagy is a lysosome-dependent intracellular degradation system that limits cellular damage and maintains homeostasis to protect cells from intra- and extracellular stress by eliminating damaged proteins and organelles^21^. It is regulated by a conserved set of proteins called autophagy-related (ATG) proteins; knockout of key ATG components, like ATG5 and ATG7, is associated with impaired autophagy, accumulation of ubiquitinated proteins, and enhanced sensitivity to stresses, such as reactive oxygen species and nutrient deficiency^22,23^. Collectively, the inhibited autophagic response due to MeHg exposure is an increasing concern. We and others have shown that MeHg activates autophagy, which prevents cell injury by MeHg^24,25^. Besides ATG genes, sequestosome 1 (SQSTM1 or p62; hereafter referred to as p62) is a vital autophagy receptor that delivers substrates to autophagosomes. p62 recognizes ubiquitinated proteins and aggregated polyubiquitinated cargoes, targeting them for lysosomal degradation^26^. We previously demonstrated the importance of p62 in MeHg detoxification. Loss of p62 in mouse embryonic cells enhances MeHg toxicity due to the impaired degradation of MeHg-induced ubiquitinated proteins, which in turn increases MeHg-derived endoplasmic reticulum stress^27,28^. Although a report showed that iHg exposure in rats led to reduced renal autophagy^29^, little is known about the relationship between iHg and autophagy. Moreover, it remains unknown whether iHg induces autophagy and whether iHg-induced autophagy differs from that induced by MeHg.

The aim of this study is to examine the autophagic response of iHg and the differences between MeHg and iHg using the MerB-expressing cells. To achieve this, we investigated the impact of MeHg or iHg on ATG expression and evaluated the activation of autophagy in wild-type (WT) and MerB-expressing cells upon MeHg exposure. Additionally, we examined the effects of iHg on the levels and intracellular localization of p62 and ubiquitinated proteins and elucidated cell viability. Herein, we show that degradation of ubiquitinated proteins via activation of autophagy and aggresome formation are characteristic cellular responses to iHg exposure.

## Results

### Exposure to MeHg and iHg increases autophagy-related gene expression

We previously demonstrated the high toxicity of intracellularly produced iHg derived from MeHg, where approximately > 60% of MerB-expressing cells survived after treatment with 4 µM MeHg for 24 h^17^. In addition, preliminary experiments revealed that treatment with 2 µM MeHg resulted in marginally increased LC3B and p62 mRNA expression in WT cells. Therefore, 4 µM MeHg was used in this study.

**Figure 2:**
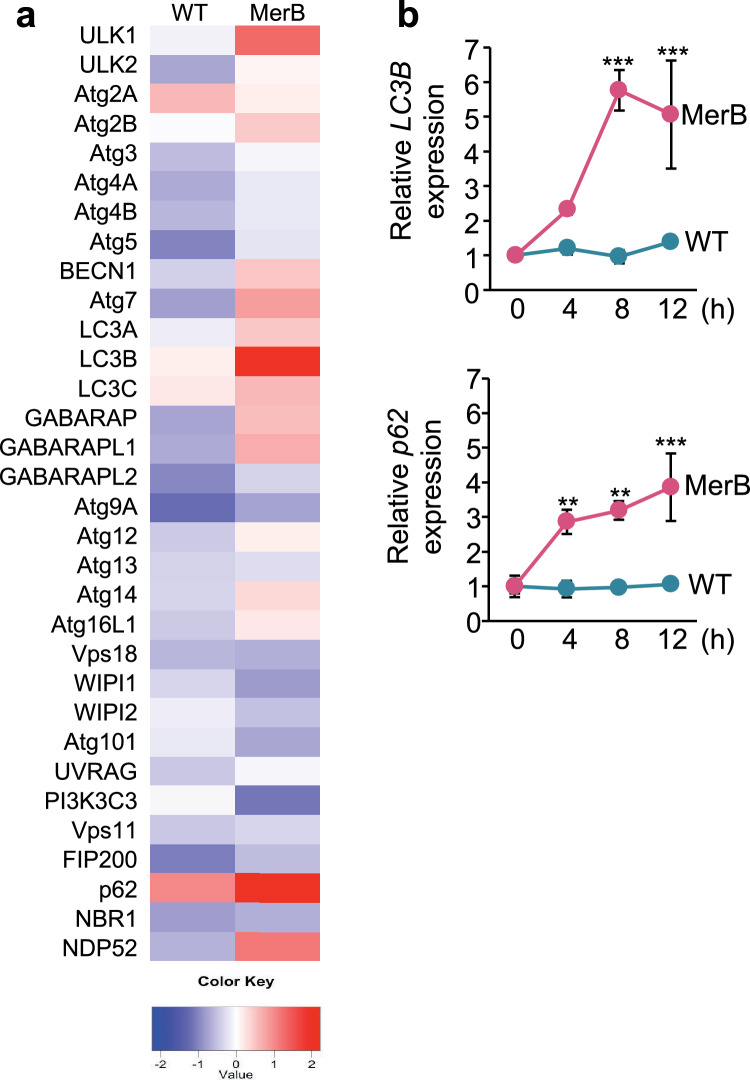
Upregulation of LC3B and p62 genes in MeHg-exposed MerB-expressing cells. (**a**) Heat map of RT-qPCR data showing gene expression (mean fold change, n = 3/treatment group) in WT and MerB-expressing cells after 8 h of treatment with 4 µM of MeHg was created using R software (ver. 4.0.5, https://www.r-project.org/). All gene values were normalized to that of the housekeeping gene, GAPDH. The color scales indicate the log 2 ratio of relative expression levels. (**b**) WT and MerB-expressing cells were treated with 4 µM MeHg and harvested at the indicated times. *LC3B* and *p62* mRNA levels were quantified by RT-qPCR. Data shown are the mean ± standard deviation (calculated from three independent experiments, each with two RT-qPCR replicates per condition; results are represented as the mean ± standard deviation values compared to DMSO-treated controls for each time point). Significant differences were determined by one-way ANOVA with multiple comparisons corrected by Dunnett test; ***p < 0.001, **p < 0.01.

**Figure 3:**
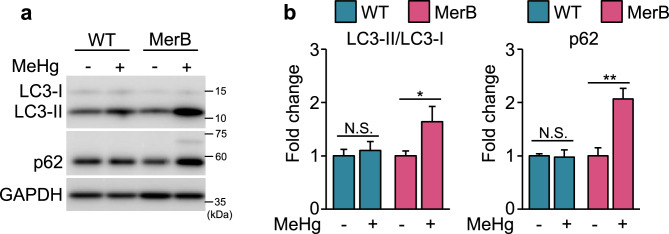
Upregulation of LC3-II and p62 proteins in MeHg-exposed MerB-expressing cells. (**a**) WT and MerB-expressing cells were treated with 4 µM MeHg for 24 h and the total lysates were subjected to immunoblotting with the indicated antibodies. Immunoblot images (a) and intensities (**b**) of WT and MerB-expressing cells were acquired as described in the methods section. The data are the average of three independent experiments, and error bars represent the standard deviations. Two-group comparisons were performed with Welch’s two sample *t*-test; N.S., not significant, **p < 0.01, *p < 0.05.

To explore the alterations in autophagy-related gene expression caused by MeHg and iHg, we treated WT human embryonic kidney cells 293 (HEK293) cells and MerB-expressing cells with 4 µM MeHg for 8 h, followed by quantitative PCR analysis. The changes in gene expression are represented as a heat map and the values of log twofold change compared to the controls of each gene are indicated by the colored scale, with red indicating a value > 1 and blue indicating a value < 1 (Fig. 2a). Consistent with our earlier findings^24^, exposure of WT cells to MeHg led to marginally higher expression levels of *microtubule-associated protein light chain 3* (*MAP1LC3/LC3)* and *p62/sequestosome-1 (p62)—*well-known autophagosome biogenesis and autophagic cargo proteins, respectively*—*but lower expression of most autophagy-related genes than that in the control cells. In contrast, MerB-expressing cells showed an increased expression of autophagy-related genes, including *LC3B, p62* (one–twofold), *unc-51-like kinase 1* (*ULK1*), *autophagy-related gene 7* (*ATG7*), GABA_A_-receptor-associated protein-like 1 (*GABARAPL1*), and *nuclear dot protein 52 kDa* (*NDP52*) (0.5–onefold). While the WT cells exhibited only minor upregulation of LC3B and p62 expression upon MeHg treatment in this study, MerB-expressing cells exhibited enhanced induction of LC3B and p62 expressions at 4 h, which was further increased at 8 and 12 h (Fig. 2b). LC3 and p62 are crucial proteins involved in both autophagosome formation and autophagy cargo recruitment^30^, implying that iHg is a potent autophagy inducer. Upon MeHg treatment, the abundance of LC3-II—a membrane-bound lipidated form of LC3^31^—and p62 was higher in MerB-expressing cells than in WT cells (Fig. 3a,b). Comparable results were obtained in MerB-expressing HeLa cells (Supplementary Fig. S1).

### iHg activates autophagic flux

**Figure 4:**
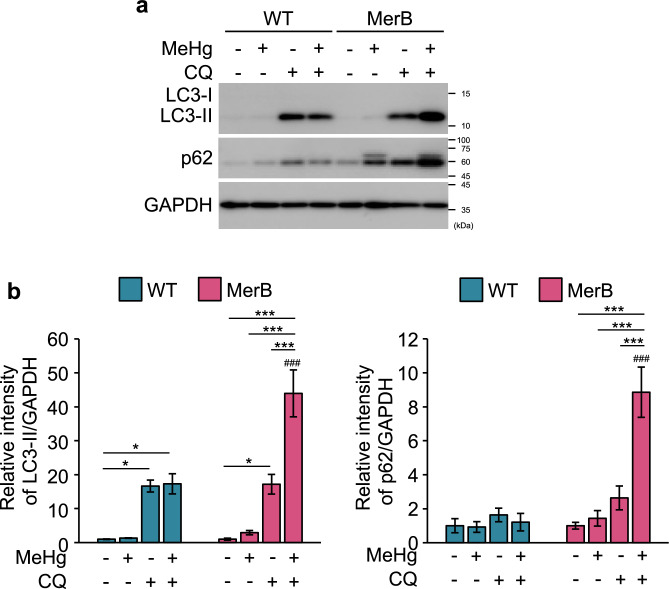
Activation of autophagic flux in MeHg-exposed MerB-expressing cells. WT and MerB-expressing cells were treated with 4 µM MeHg in the presence or absence of 20 µM chloroquine (CQ) for 24 h. (**a**) The total lysates were subjected to immunoblotting with the indicated antibodies. (**b**) The bar graphs represent data from replicated experiments (*n* = 3, Tukey’s HSD ***p < 0.001, *p < 0.05; ^###^*p* < 0.001 versus WT).

The determine whether the iHg-induced upregulation of LC3-II protein resulted from activation of autophagy or the blockade of downstream autophagic degradation steps^32^, we treated WT and MerB-expressing cells with 4 µM MeHg in the absence or presence of the lysosomal inhibitor chloroquine (CQ) and evaluated the autophagic flux. Immunoblot analyses revealed that LC3-II protein levels were higher in cells co-treated with MeHg and CQ than in those treated with MeHg or CQ alone (Fig. 4a,b). Moreover, p62 protein levels were elevated in MerB-expressing cells following MeHg treatment and further increased in CQ co-treated cells, indicating that p62 was degraded during iHg-induced autophagy. Conversely, no marked difference in LC3-II or p62 protein levels were observed in WT cells treated with MeHg or CQ alone or together. These findings suggest that autophagic flux in response to iHg is higher than that in response to MeHg.

### iHg induces ubiquitinated protein aggregate (aggresome) formation

**Figure 5:**
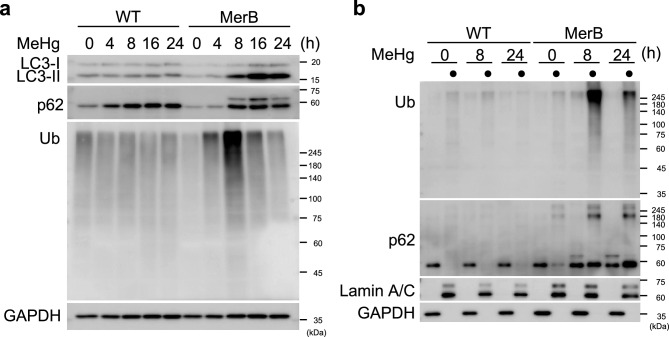
Accumulation of ubiquitinated protein aggregates in MeHg-exposed MerB-expressing cells. (**a**) WT and MerB-expressing cells were treated with 4 µM MeHg for the indicated periods. Cells were harvested, and the total lysates were subjected to immunoblotting with the indicated antibodies. (**b**) WT and MerB-expressing cells were treated with 4 µM MeHg for the indicated periods and then treated with 1% Triton X-100 for 15 min at 4 °C. Solubilized proteins were removed by centrifugation. Soluble and insoluble (•) fractions were subjected to immunoblotting with the indicated antibodies.

**Figure 6:**
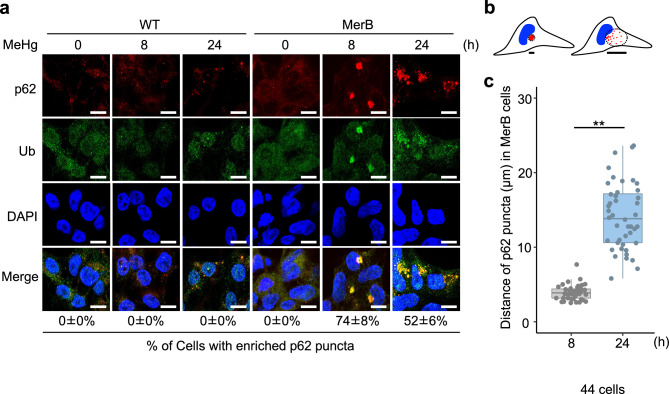
Transient formation of large p62 and ubiquitin-positive aggregates in MeHg-exposed MerB-expressing cells. (**a**) WT and MerB-expressing cells were treated with 4 µM MeHg for the indicated periods, and then immunofluorescence staining was performed with p62 and ubiquitin antibodies. Scale bar, 20 µm. (**b**) Graphics outlining the scheme for measuring the dispersion distance of p62 in the Fig. 6c experiment. (**c**) Boxplot showing the dispersion distance of p62 from confocal images of MerB-expressing cells. *n* = 44 cells (MeHg for 8 h) and 44 cells (MeHg for 24 h). In the boxplot, the center line indicates the median, and box boundaries indicate the 25th and 75th percentiles; **, *p* < 0.01 (Bunner–Munzel test).

The primary role of autophagy is to remove harmful substances, including damaged organelles and protein aggregates, from the cell. Our previous study demonstrated that MeHg leads to the accumulation of ubiquitinated proteins in mouse embryonic fibroblast (MEF) cells^27^. Therefore, in this study, we investigated the accumulation of ubiquitinated proteins by iHg. We found that the accumulation of ubiquitinated proteins in MerB-expressing cells upon MeHg treatment was higher than that in WT cells (Fig. 5a). The accumulation of ubiquitinated proteins in MerB-expressing cells peaked 8 h after MeHg treatment and subsequently declined. In contrast, the accumulation of ubiquitinated proteins was not discernible in WT cells treated with MeHg. Transient accumulation of ubiquitinated proteins and p62 in MerB-expressing cells after MeHg treatment was detected in the TritonX-100 insoluble fractions (Fig. 5b). Similar results were obtained in MerB-expressing HeLa cells (Supplementary Fig. S2a,b). To determine the status of ubiquitinated proteins and p62 in MerB-expressing cells following MeHg treatment, we conducted an immunofluorescence analysis. Large aggregates (aggresomes) containing ubiquitinated protein and p62 were formed in MerB-expressing cells 8 h after MeHg treatment (Fig. 6a). These aggresomes were fragmented into smaller aggregates after 24 h of MeHg treatment (Fig. 6a,b). In contrast, aggresomes were not observed in WT cells following MeHg treatment, indicating that iHg promotes aggresome formation.

### Autophagy attenuates iHg-induced cytotoxicity

**Figure 7:**
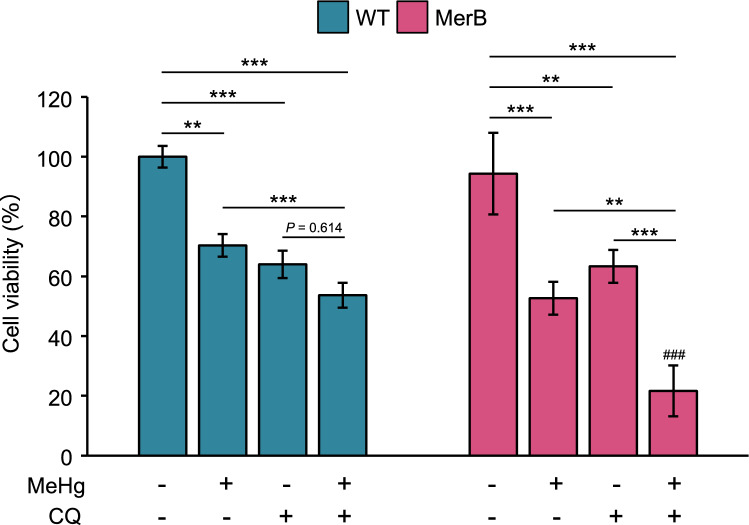
Promotion of iHg-mediated cell death by CQ. WT and MerB-expressing cells were exposed to 4 µM MeHg in the presence or absence of 20 µM CQ for 24 h. Cell viability was determined using a CCK-8 assay. Cell viability of WT and MerB-expressing cells. The bar graph represents data from replicated experiments (*n* = 3, Tukey’s HSD ***p < 0.001, **p < 0.01; ^###^*p* < 0.001 versus WT).

To explore the role of autophagy induced by iHg, we examined the effect of autophagy inhibition by CQ on iHg toxicity (Fig. 7). The cell viability of WT and MerB-expressing cells after treatment with MeHg in the presence or absence of CQ for 24 h was measured by CCK-8 assay. A substantial decrease in the cell viability of MerB-expressing cells was observed upon combined treatment with MeHg and CQ compared to MeHg or CQ alone (Fig. 7). In contrast, there was no significant difference in the cell viability of WT cells upon combined treatment with MeHg and CQ compared to CQ alone. Intriguingly, the total concentrations of Hg or iHg in WT and MerB-expressing cells were largely unaffected by CQ (Supplementary Fig. S3a,b), indicating that the reduction in cell viability of MerB-expressing cells by the combined treatment of MeHg and CQ was not due to the accumulation of total Hg or iHg. Hence, this reduced cell viability following MeHg and CQ treatment suggests that autophagy, in part, plays a significant role in preventing cells from iHg-induced cytotoxicity.

## Discussion

In this study, we investigated autophagy using MerB-expressing cells—useful model systems that overcome the low cell permeability of iHg—to investigate the effect of iHg on intracellular systems. We showed that iHg activates autophagy, leading to dramatic changes in the protein expression levels of autophagy markers such as LC3-II and p62. Further investigations revealed, for the first time, that iHg promotes aggresome formation. Moreover, inhibition of autophagy by CQ resulted in decreased cell viability of MeHg-exposed MerB-expressing cells. Therefore, our findings indicate that aggresome formation and autophagy activation serve as key protective mechanisms against iHg-mediated toxicity.

The ULK1 complex, comprising ULK1 along with ATG13, FIP200, and ATG101, is a crucial player in autophagosome formation. Phagophore expansion is facilitated by ATG7, which enhances ATG8 (LC3 and GABARAP families) lipidation. The lipidated form of LC3 (LC3-II) promotes phagophore expansion and autophagosome formation^33^, while p62 and NDP52 bind to ubiquitinated substrates and aid in the delivery of substrates to the autophagosome, suggesting that the iHg derived from MeHg in MerB-expressing cells activates many genes that promote autophagy. Moreover, the upregulation of LC3-II and p62 in MerB-expressing cells after treatment with MeHg (Fig. 3a,b, Supplementary Fig. S1) supports the notion of autophagy promotion by iHg. In contrast, MeHg treatment of WT cells led to a marginal increase in LC3-II and upregulated p62 protein levels. Total intracellular concentrations of Hg did not differ significantly between the two cell lines (i.e., WT cells and MerB-expressing cells; Supplementary Fig. S3a), suggesting that the enhanced effects on autophagy are mainly caused primarily by iHg itself and not by MeHg.

What is the key mechanism underlying iHg-induced activation of autophagy? Here, we provide initial data suggesting that the activation of iHg-induced autophagy may be due to increased activity of the transcription factor Nrf2. Nrf2 functions as a transcription factor when the cysteine residue of Keap1 is modified, thus hindering the bond between Nrf2 and Keap1. Our results have demonstrated Nrf2 is markedly activated in MerB-expressing cells upon MeHg treatment compared to WT cells (Supplementary Fig. S4a,b). According to our previous experiments, when MerB-expressing cells are treated with the antioxidant Trolox, the expression of heme oxygenase 1—a target gene of Nrf2—is negligibly affected^17^. Therefore, the activation of Nrf2 is not likely mediated by reactive oxygen species (ROS) but results from the direct binding of iHg to cysteine residues in Keap1. Meanwhile, the phosphatidylinositol 3-kinase (PI3K)/Akt pathway, which is essential for regulating autophagy, can be promoted in response to ROS^34^. In our previous study, higher ROS levels were observed in MerB-expressing cells treated with MeHg than in WT cells^17^. Although the mechanism underlying autophagy activation is unclear, given the effect of Trolox, we postulate that PI3K/Akt activation is suppressed; thus, Nrf2 activation, more than PI3K/Akt, contributes to autophagy activation.

Upon autophagy inhibition, autophagosomes accumulate due to the prevention of lysosomal/autophagosomal fusion, resulting in increased levels of autophagosomal LC3-II and ubiquitin receptor p62^35^. Thus, autophagic flux can be monitored by measuring the expression levels of LC3-II and p62 in the presence or absence of autophagy inhibitors. Consistent with previous studies^36,37^, we observed higher levels of LC3-II in MerB-expressing cells upon combined treatment with MeHg and CQ than with CQ alone, indicating an activation of autophagy (Fig. 4a,b). Similar to LC3, western blot analysis of p62 in the presence or absence of CQ provides essential information about the activation of autophagic flux, suggesting that iHg activates autophagic flux. Moreover, CQ did not induce an increase in both LC3-II or p62 induction in WT cells, indicating that this concentration of MeHg did not induce autophagy in HEK293 cells. In our previous study, we showed that MeHg activates autophagy in several cell lines, including SH-SY5Y and MEF cells^24^. Meanwhile, in the current study, MeHg did not induce autophagy in HEK293 cells. This may be due to HEK293 cells being less sensitive to MeHg than to SH-SY5Y and MEF cells; therefore, 4 µM MeHg had minimal effects on autophagy. Since the autophagy response is triggered by iHg production in MerB-expressing cells, treating WT HEK293 cells with > 4 µM MeHg likely induces the autophagy response. Collectively, these considerations suggest that iHg drives autophagy more effectively than MeHg.

During autophagy, p62 contributes to the formation of ubiquitinated protein aggregates, which include damaged proteins or dysfunctional organelles, to mitigate stress and maintain cellular homeostasis^38^. This protein has been identified as a component of ubiquitinated protein aggregates found in individuals suffering from neurodegenerative diseases, such as Alzheimer’s disease, Parkinson’s disease, and Huntington disease^39,40^. Our findings indicate that there are transiently high levels of p62 and ubiquitinated proteins in MerB-expressing cells following treatment with MeHg compared to WT cells (Fig. 5a, Supplementary Fig. S2a,b). This suggests that iHg but not MeHg may strongly induce ubiquitinated proteins. In addition, detergent extraction of MerB-expressing cells revealed that large amounts of ubiquitinated proteins and p62 were not dissolved by 1% Triton X-100 (Fig. 5b), implying that iHg-induced ubiquitinated proteins form aggregates with p62^41^. Consistently, iHg-induced ubiquitinated proteins were found to co-localize with p62, forming large aggregates in the perinuclear region (Fig. 6a), resembling features of “aggresome” or “aggresome-like induced structure (ALIS).” These aggregates are recognized as a cytoprotective response that help reduce proteotoxic stress^42,43^ and require p62 expression^44,45^. Considering that p62 upregulation is highly dependent on the activation of Nrf2, we believe that iHg-induced formation of aggresomes/ALIS is likely more dependent on the activation of Nrf2 by iHg than on the generation of ROS. However, the involvement of excess ROS in iHg-induced production of ubiquitinated proteins may also induce aggresome/ALIS formation, which is a subject for future studies.

Our immunoblot and immunofluorescence analyses also demonstrated that reducing the level of ubiquitinated protein correlated with fragmentation of aggresome/ALIS structures into small aggregates in MerB-expressing cells (Figs. 5 and 6). These results suggest the possible involvement of the protein degradation system in iHg-induced ubiquitinated protein clearance in MerB-expressing cells. Generally, these aggregates are thought to be degraded by autophagy, facilitating clearance^46^. Our cell viability profiles using CQ showed that blocking autophagy promoted iHg toxicity (Fig. 7), implying that the aggresome/ALIS–autophagy pathway plays a cytoprotective role against iHg toxicity.

However, there are certain limitations to this study. First, we investigated autophagic responses using engineered artificial cell models expressing bacterial demethylation enzymes, which may not fully reflect the in vivo environment. It should be noted that iHg produced in vitro may be highly cytotoxic and not effectively excreted from cells. Therefore, we cannot rule out that the in vitro cellular response may differ from the cellular response when Hg^2+^ is generated from MeHg in vivo. Ultimately, we believe our study findings and their relevance to MeHg can be discussed by determining the mechanism of physiological MeHg demethylation. Second, our previous report revealed that MerB is expressed in the cytoplasm, suggesting that the demethylation of MeHg occurs in the cytoplasm of MerB-expressing cells. However, the physiological subcellular localization of this conversion remains unknown and may activate other signaling pathways distinct from MerB-expressing cells. Third, despite comparison with WT cells, the combined effect of iHg and MeHg cannot be excluded. Moreover, it is unclear whether the results reflect the toxicity of iHg converted from MeHg exposed to environmental contamination. The relevance of the results of this study with respect to disorders arising from MeHg toxicity, such as Minamata disease, is unclear. Nevertheless, even with these limitations considered, MerB-expressing cells provide an opportunity to investigate the intracellular effect of iHg without exposing cells to excessive extracellular concentrations. Therefore, MerB-expressing cells may offer valuable insights into deciphering the protective responses against intracellular iHg-mediated toxicity.

In conclusion, this study has overcome the difficulties of examining iHg-dependent cellular responses using MerB-expressing cells and demonstrates that iHg strongly activates autophagy and facilitates aggresome formation, defending the cells against iHg toxicity. Our results provide novel insights into the cellular responses against iHg and the possibility of developing anti-iHg drugs owing to the autophagy induction and aggresome formation in iHg-containing cells.

## Methods

### Antibodies and reagents

Antibodies against p62, ubiquitin, and glyceraldehyde 3-phosphate dehydrogenase (GAPDH) were purchased from Cell Signaling Technology (Beverly, MA, USA). Horseradish peroxidase (HRP)-conjugated secondary antibodies were purchased from GE Healthcare (Buckinghamshire, UK). Methylmercury chloride (Tokyo Kasei, Tokyo, Japan) was dissolved in dimethyl sulfoxide (DMSO) and maintained as a 25 mM stock solution. A stock solution of chloroquine diphosphate salt (100 mM; Sigma-Aldrich, St. Louis, MO, USA) was prepared in water and sterilized using a 0.22 µm pore size hydrophilic polyvinylidene fluoride (PVDF) membrane (EMD Millipore, Billerica, MA, USA).

### Cell culture and methylmercury chloride treatment

We previously established MerB-expressing HEK293 and HeLa cell lines^17^, which were used in this study. All experiments were performed and analyzed in accordance with regulations for the genetic modification experiments at Kitasato University. Cells were maintained in Dulbecco’s modified Eagle’s medium (DMEM) supplemented with 10% heat-inactivated fetal bovine serum (Tissue Culture Biologicals, Seal Beach, CA, USA), 100 U/mL penicillin, 100 µg/mL streptomycin, and 292 µg/mL L-glutamine (Thermo Fisher Scientific, Rockford, IL, USA). Cells were maintained at 37 °C in a 5% CO_2_ humidified atmosphere. Cells were treated with different concentrations of MeHg or DMSO, which served as a control. In the autophagic flux or cell viability assays, cells were treated with MeHg in the presence or absence of 20 µM CQ for 24 h.

### Immunoblot analysis

Cells were lysed with radioimmunoprecipitation assay (RIPA) buffer (20 mM Tris pH 7.4, 0.1% sodium dodecyl sulfate (SDS), 1% sodium deoxycholate, 1% NP40, and protease/phosphatase inhibitor cocktail (Cell Signaling Technology, Danvers, MA, USA)), and sonicated for 10 s on ice. For separation experiments, cells were treated with Triton X-100 buffer (1% Triton X-100, 20 mM Tris pH 7.4, 137 mM NaCl, and 2 mM ethylenediaminetetraacetic acid, protease/phosphatase inhibitor cocktail) for 15 min at 4 °C, followed by centrifugation at 15,000 × g for 10 min. The detergent-insoluble fraction was solubilized in RIPA buffer. The detergent-soluble and -insoluble fractions were subjected to immunoblot analysis as previously described^27^. For nucleus/cytoplasm separation, cells were lysed with 1% Nonidet P-40 for 5 min on ice, followed by centrifugation at 228 × g for 5 min at 4 °C. The supernatants were used as the cytoplasmic fractions (C), whereas the detergent-insoluble fractions were used as nucleus fractions (N)^47^. To prepare samples for immunoblot analysis, 10 µg of protein were taken from cell lysates or fraction solutions and mixed with Laemmli buffer. Samples were then heated at were at 95 °C for 5 min. Afterward, the samples were separated using SDS polyacrylamide gel electrophoresis (SDS-PAGE), and the proteins were electrotransferred to PVDF membranes (EMD Millipore). The membranes were treated with tris-buffered saline (TBS) containing 0.1% (w/w) Tween 20 (TTBS) and 5% (w/v) skim milk and incubated at 4 °C overnight with the corresponding primary antibodies in a blocking solution (1:1000). The following day, membranes were further incubated with HRP-conjugated secondary antibodies (1:6000). The protein blots were visualized using a clarity-enhanced chemiluminescence reagent (Nakalai Tesque, San Diego, CA, USA) in an Amersham Imager 680 (GE Healthcare) and quantified using the ImageQuant TL 8.1 image analysis software (GE Healthcare). If staining with additional antibodies was required, the membranes were blocked with skim milk, and the staining was repeated.

### Confocal microscopy

Confocal microscopy image analysis was conducted as previously described^27^ with modifications. Cells were grown in six-well plates on glass coverslips with rat tail collagen I (Neuvitro Corporation, Vancouver, WA, USA). Following treatment, cells were fixed with 4% paraformaldehyde in phosphate-buffered saline (PBS) at 25 °C for 10 min. They were then transferred to a membrane permeabilization solution (0.2% Triton X-100) for 10 min. Cells were blocked in PBS that contained 1% bovine serum albumin for 1 h. For p62 and ubiquitin staining, cells were incubated with anti-p62 (1:400) and anti-ubiquitin (1:200) antibodies at 25 °C for 1.5 h. Subsequently, cells were treated with appropriate secondary antibodies labeled with Alexa Fluor Plus 488 or 555 (Thermo Fisher Scientific, 1:1000) for 30 min. After three washes with PBS, cells were mounted on the slide using ProLong Glass Antifade Mountant with NucBlue Stain (Thermo Fisher Scientific). Immunofluorescence was visualized using an FV3000 confocal laser scanning microscope (Olympus, Tokyo, Japan). Images were processed using the FV3000 system software (Olympus, FV31S-SW).

### Reverse transcription-polymerase chain reaction (RT-PCR)

Total RNA was extracted from cells after treatment with MeHg using a NucleoSpin RNA kit (Macherey–Nagel, Bethlehem, PA, USA) and reverse-transcribed using PrimeScript RT Master Mix (Thermo Fisher Scientific) to generate cDNA as described previously^17^. Each real-time PCR reaction consisted of 1 µL of cDNA template, 5 µL of PowerUp SYBR Green Master Mix (Thermo Fisher Scientific), and 4 pmol of forward and reverse primers on the CFX-96 thermal cycler system (Bio-Rad, Hercules, CA, USA) for 40 cycles (95 °C for 2 min, 55 °C for 10 s, and 72 °C for 10 s) after an initial 2 min of incubation at 95 °C. The housekeeping gene GAPDH mRNA was used to normalize the level of cDNA. Triplicate samples were assessed for each gene of interest. The results were evaluated using Bio-Rad CFX Manager software (version 3.1; Bio-Rad). The quantification cycle (Cq) value was read, and the relative expression of the target gene was determined using the 2^-∆∆Ct^ method. The primers used are given in Supplementary Table 1.

### Cell viability assay

Cell viability was assessed via the water-soluble tetrazolium salt assay using the Cell Counting Kit-8 (CCK-8; Dojindo, Kumamoto, Japan) according to the manufacturer’s instructions. The cells were seeded onto 96-well plates (1 × 10^4^ cells/well) and treated with different conditions for 24 h. Next, 10 µL CCK-8 solution was added to the wells and the plates were incubated for an additional 1 h at 37 °C. Cell viability was assessed by measuring the absorbance at 450 nm using an iMark microplate reader (Bio-Rad).

### Mercury analysis

The content of total and inorganic mercury was measured using an MA-3 Solo Mercury Analyzer (Nippon Instruments Co., Tokyo, Japan). Cells in 60-mm dishes were washed using ice-cold PBS and then lysed in RIPA buffer. The cell lysates were used in assays for quantifying total mercury, inorganic mercury, and whole protein content. The content of inorganic mercury in each cell lysate was determined as previously described^17^.

### Statistical analysis

Cell viability and quantitative RT-PCR data were analyzed using one-way analysis of variance (ANOVA), followed by Tukey’s HSD test (*p* < 0.05). Values are presented as the mean ± standard deviation. *p*-values of < 0.05, 0.01, and 0.001 are represented as *p < 0.05, **p < 0.01, and ***p < 0.001, respectively. R software (ver. 4.0.5) and Microsoft Excel (ver. 16.48; Microsoft, Redmond, WA, USA) were used for statistical analyses.

### Supplementary Information

Supplementary Figures.

Supplementary Table 1.

Supplementary Figures.

## Data Availability

All materials and data are available upon reasonable request from the corresponding author.
