# Supplementary material for: Conversion of methylmercury into inorganic mercury via organomercurial lyase (MerB) activates autophagy and aggresome formation
Source: Sci Rep. 2023 Nov 15;13:19958. doi: 10.1038/s41598-023-47110-y (PMC10651920; doi:10.1038/s41598-023-47110-y)

Original immunoblot for three repeats (Figure 3a)

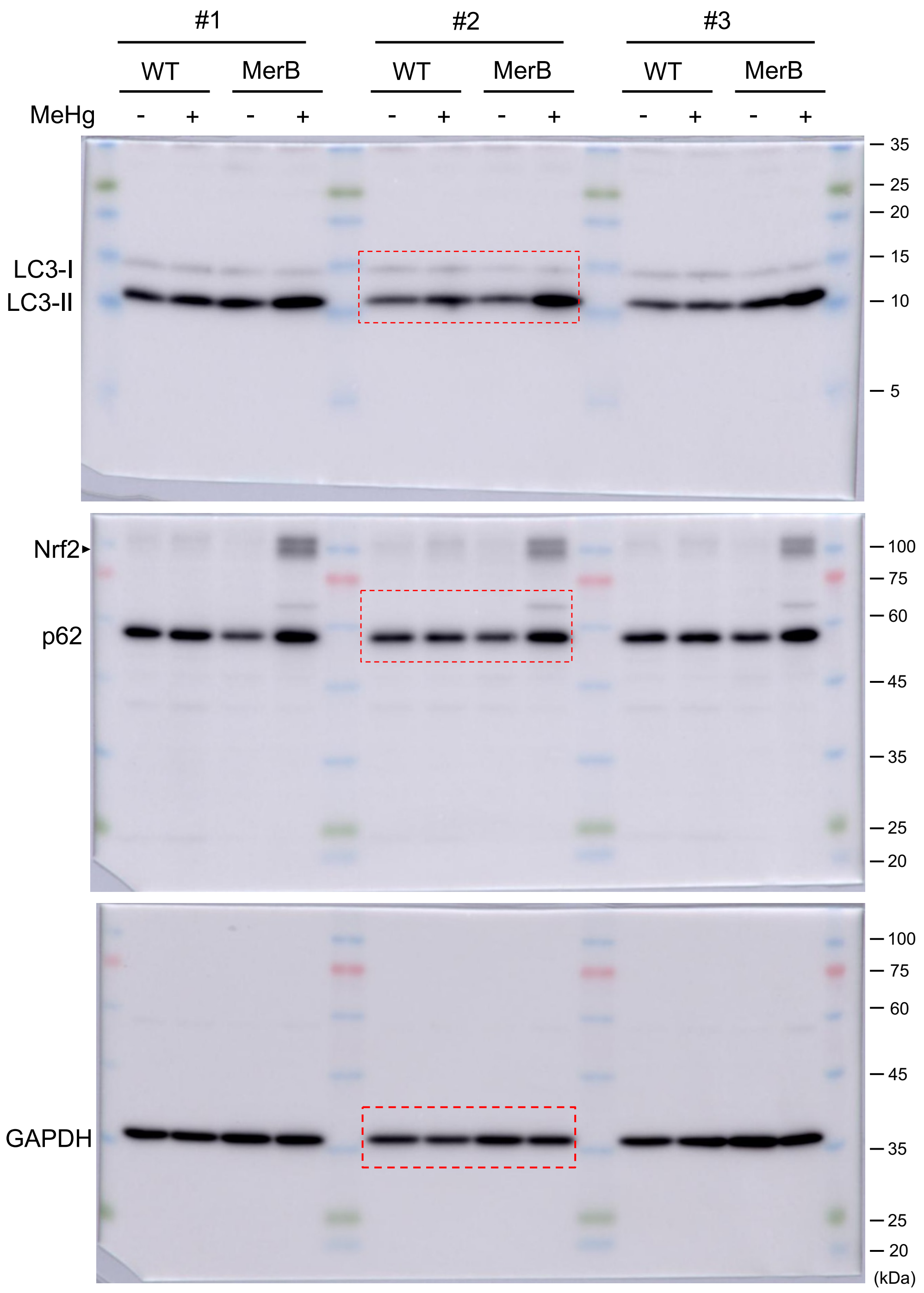

Original immunoblot (Figure 4a)

(The two gels were transferred to one PDVF membrane.)

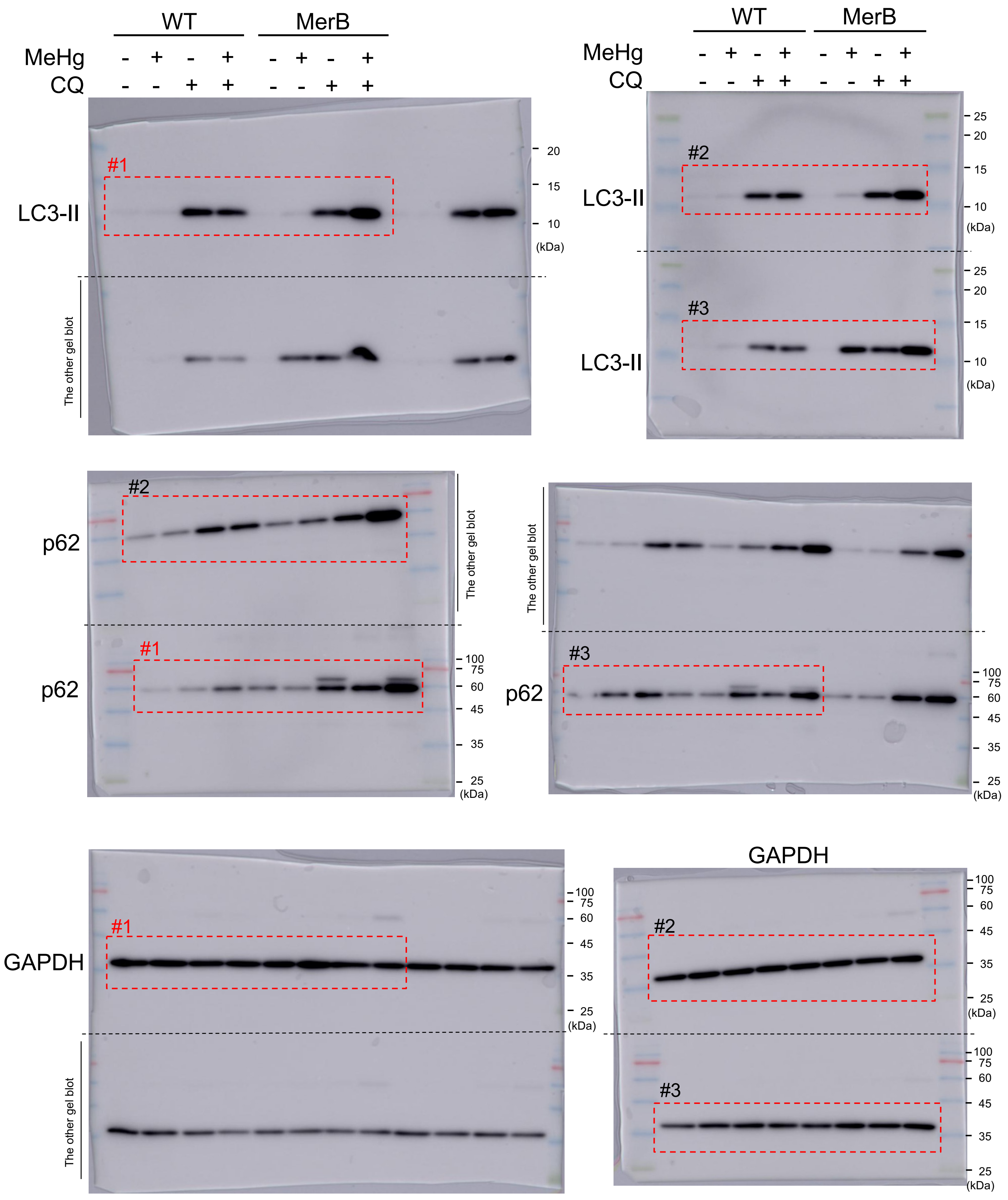

Original immunoblot (Figure 5a, b)

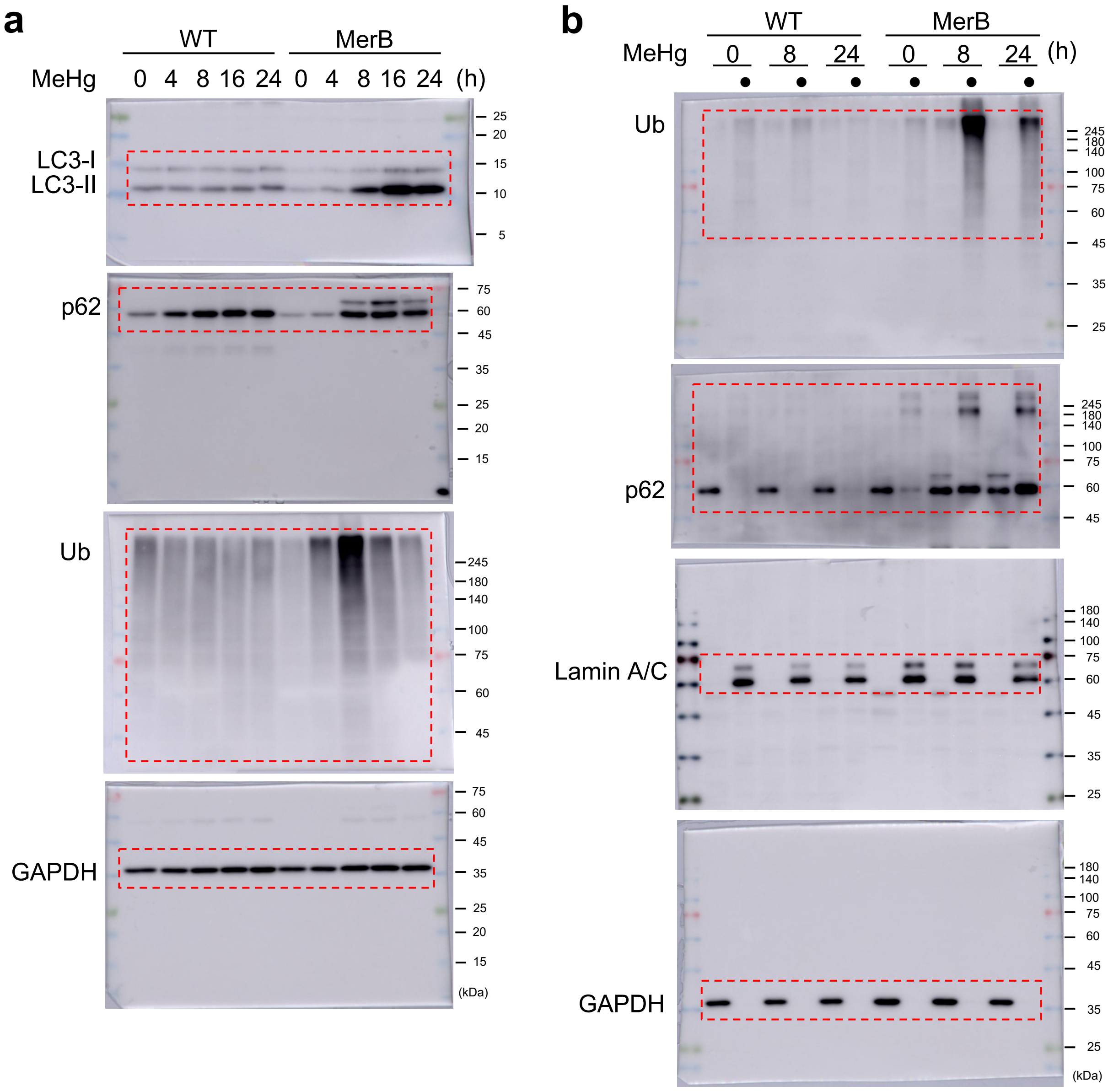

Original immunoblot (Supplementary Figure S1)

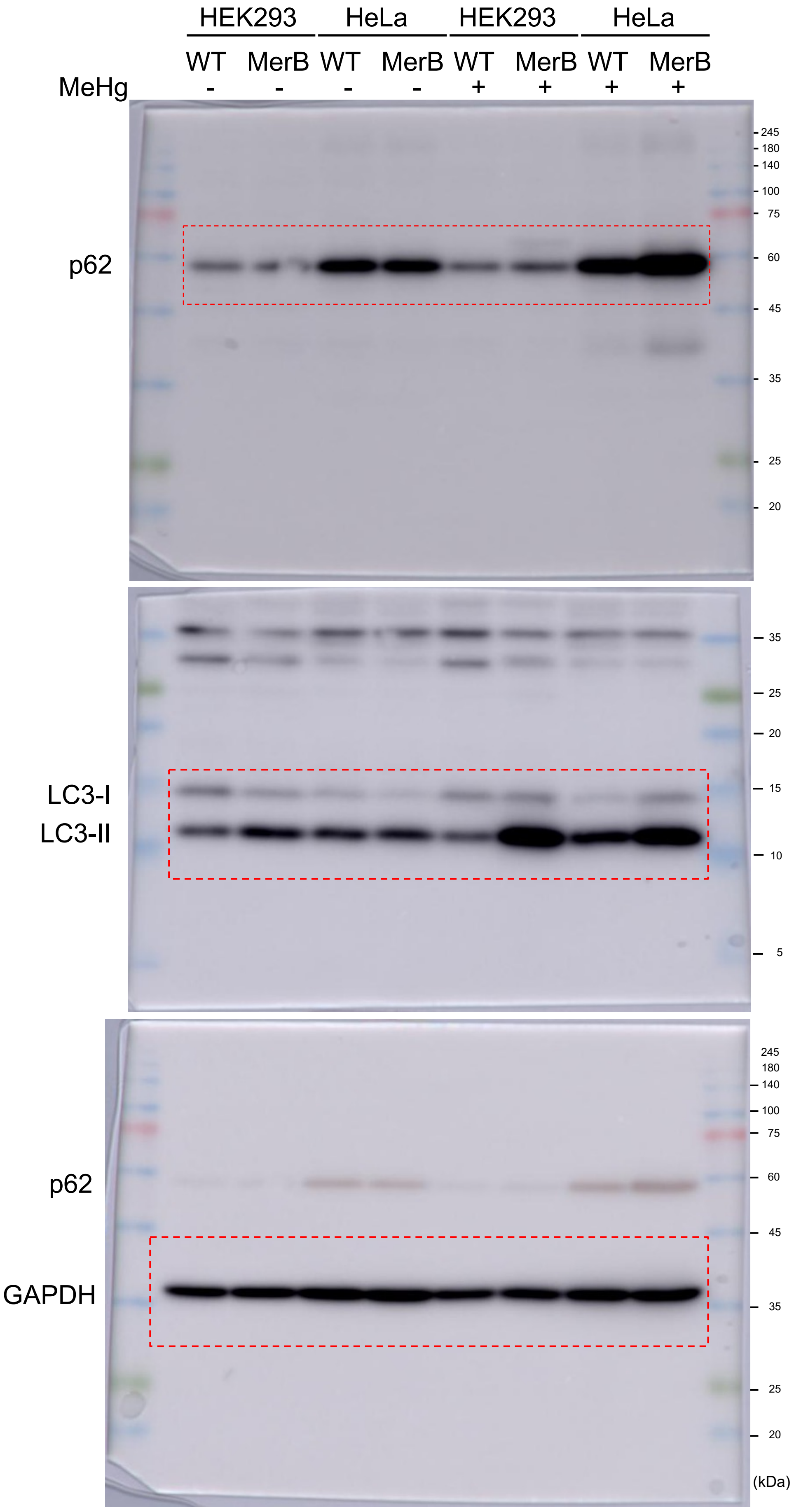

Original immunoblot (Supplementary Figure S2a, b)

a

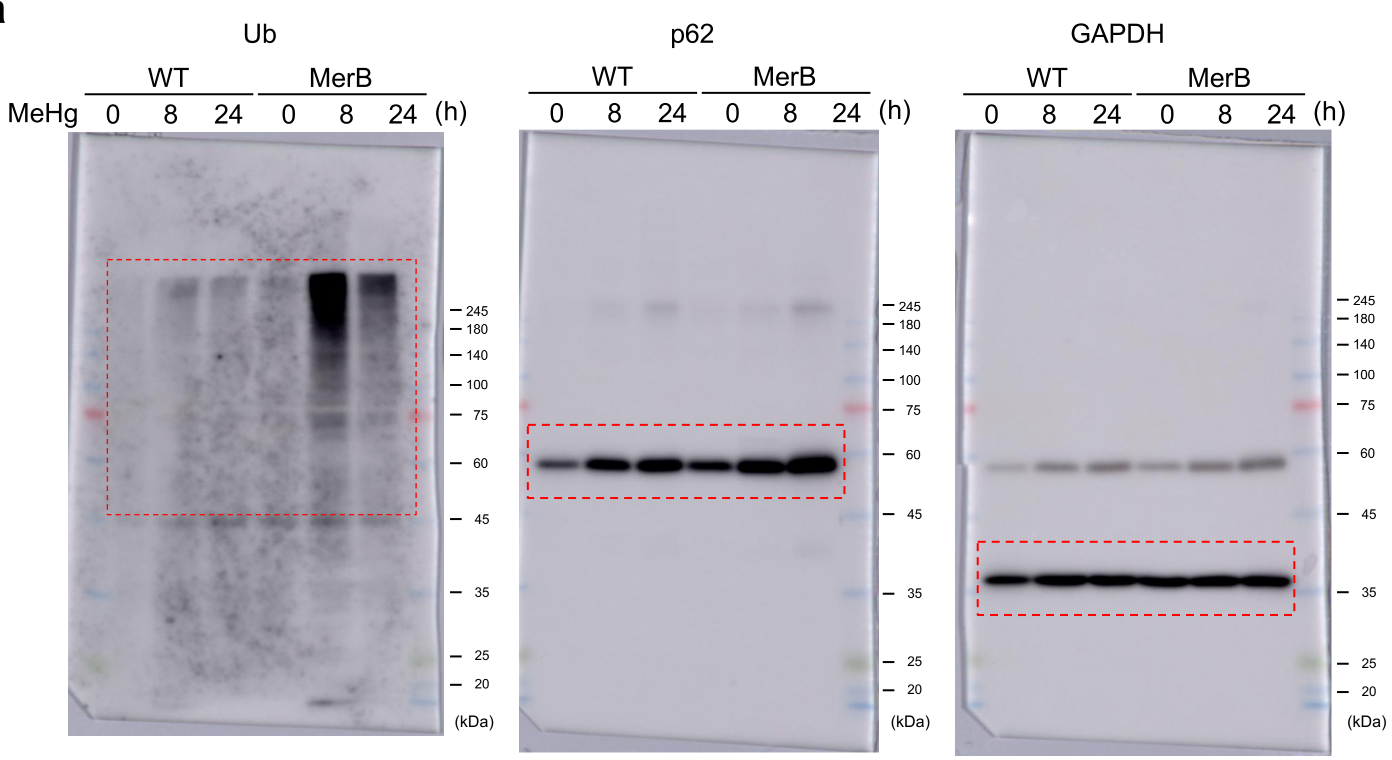

b

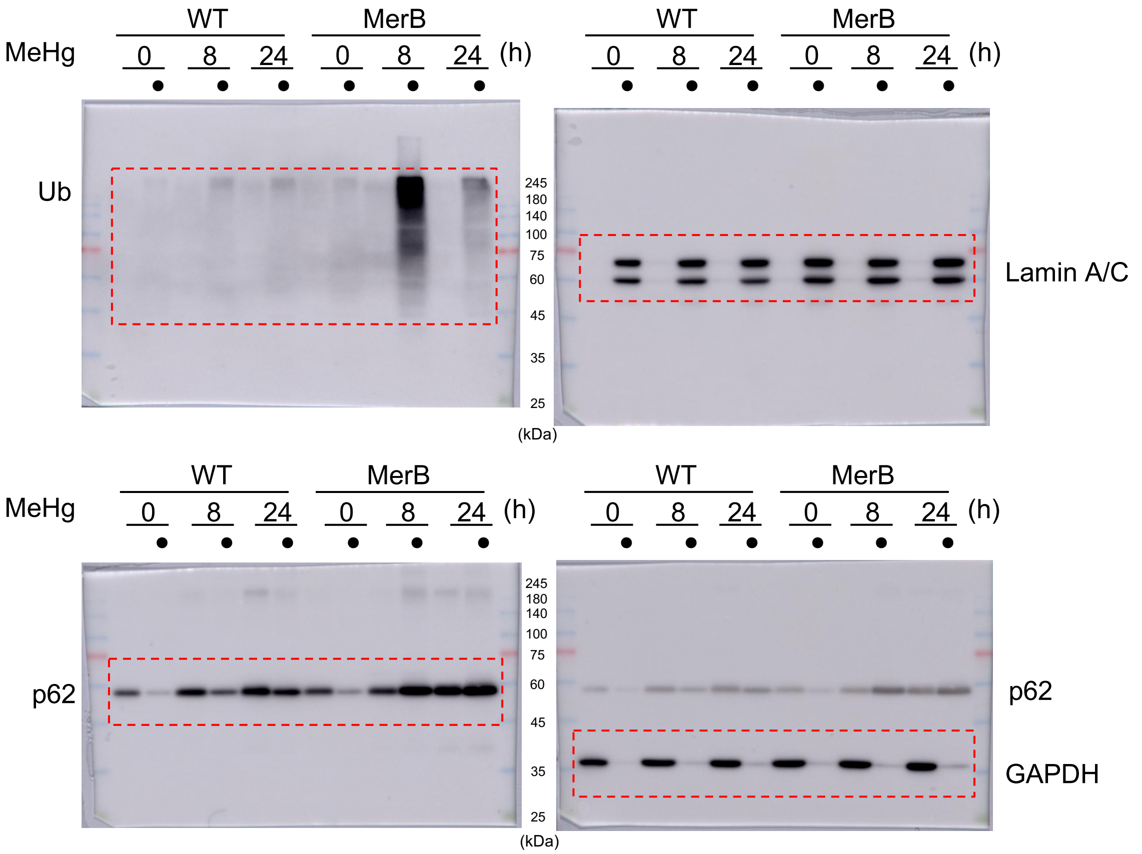

Original immunoblot (Supplementary Figure S4a, b)

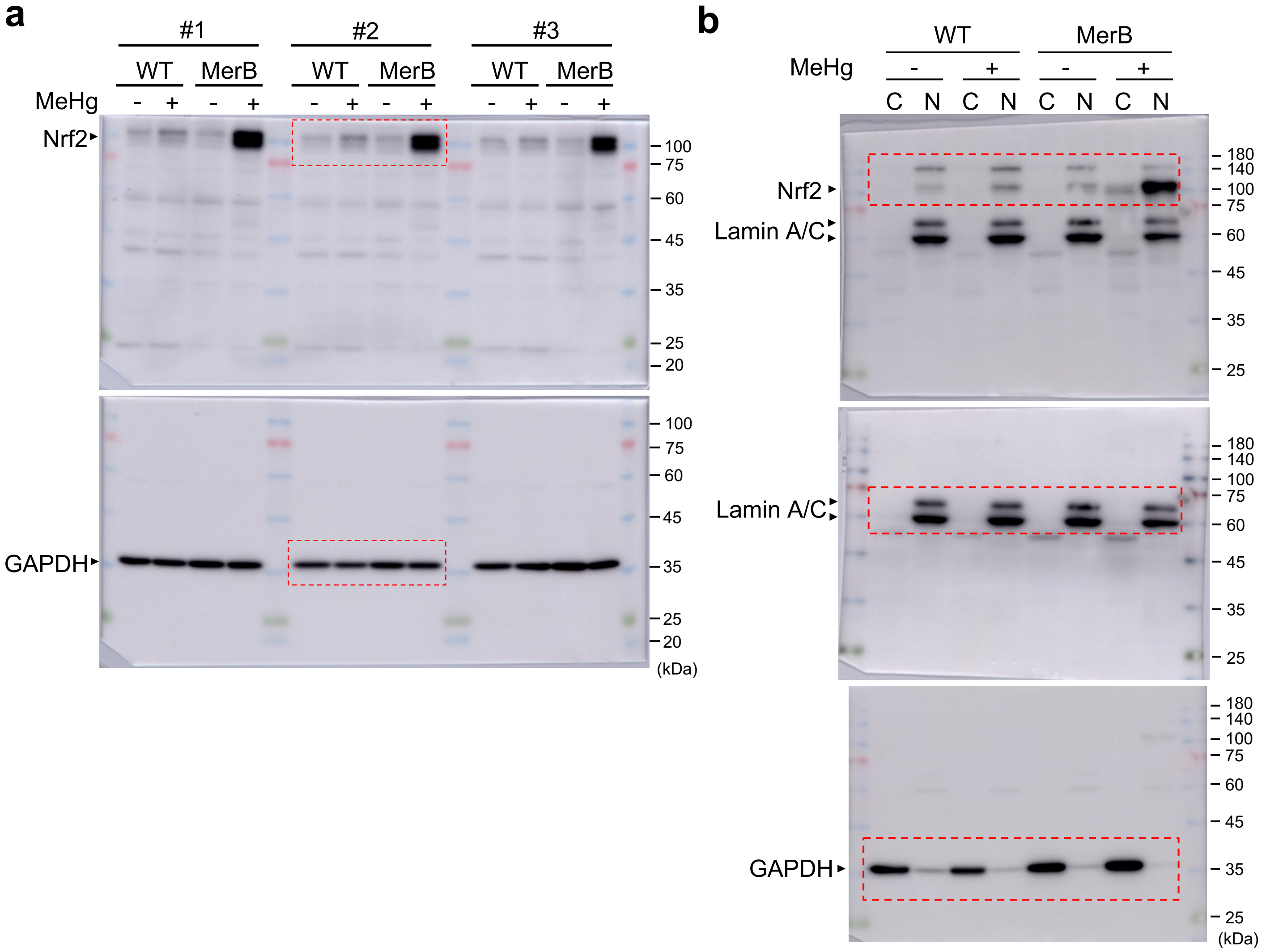

Supplement: Supplementary file 1 — Supplementary Figures. [file 41598_2023_47110_MOESM1_ESM.pdf]
