# Supplementary material for: Conversion of methylmercury into inorganic mercury via organomercurial lyase (MerB) activates autophagy and aggresome formation
Source: Sci Rep. 2023 Nov 15;13:19958. doi: 10.1038/s41598-023-47110-y (PMC10651920; doi:10.1038/s41598-023-47110-y)
Supplement: Supplementary file 2 — Supplementary Table 1. [file 41598_2023_47110_MOESM2_ESM.docx]

**Supplemental Table 1. Primer sequences for RT-PCR analysis**

**
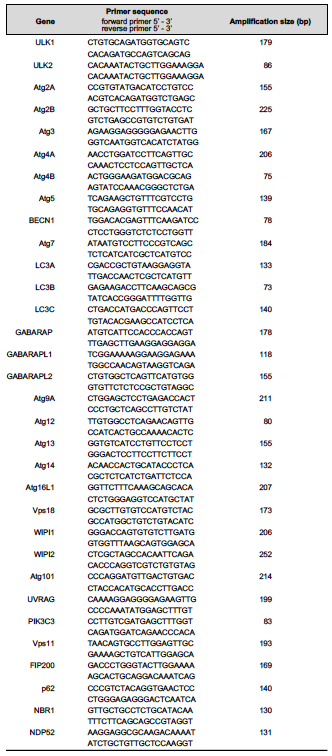
**

Abbreviations: ULK = unc-51 like autophagy activating kinase; ATG = autophagy-related protein; BECN1; LC3 = light chain 3; GABARAP = GABA(A) receptor-associated protein; GABARAPL = GABA(A) receptor-associated protein like; Vps = vacuolar protein sorting-associated protein; WIPI = WD repeat domain phosphoinositide-interacting protein; UVRAG = UV radiation resistance associated; PIK3C3 = phosphatidylinositol 3-kinase catalytic subunit type 3; FIP = focal adhesion kinase family interacting protein; NBR = neighbor of BRCA; NDP52 = nuclear dot 10 protein 52
