# Supplementary material for: Conversion of methylmercury into inorganic mercury via organomercurial lyase (MerB) activates autophagy and aggresome formation
Source: Sci Rep. 2023 Nov 15;13:19958. doi: 10.1038/s41598-023-47110-y (PMC10651920; doi:10.1038/s41598-023-47110-y)
Supplement: Supplementary file 3 — Supplementary Figures. [file 41598_2023_47110_MOESM3_ESM.docx]

**Supplementary Figure S1**

**
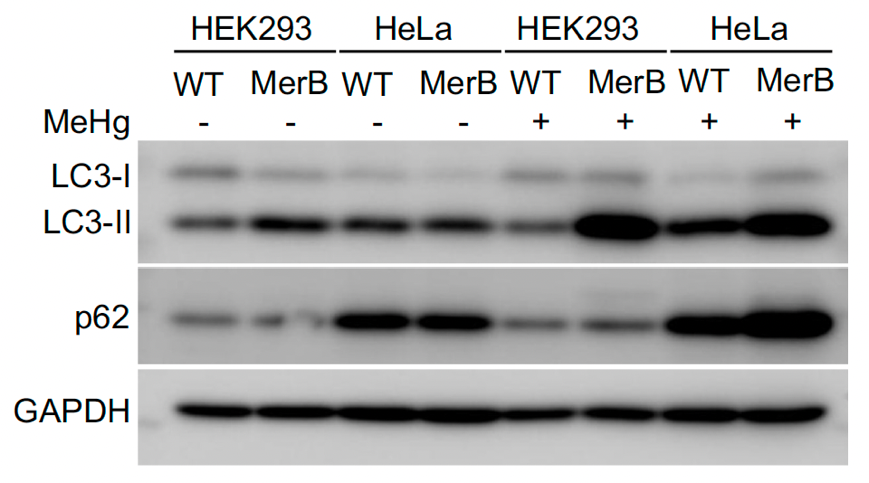
**

HEK293 or HeLa cells (WT and MerB-expressing cells) were treated with 4 µM MeHg for 24 h, and the total lysates were subjected to immunoblotting with the indicated antibodies.

**Supplementary Figure S2**

**
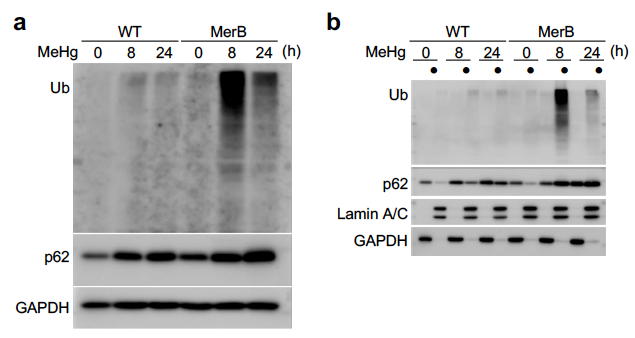
**

(a) WT and MerB-expressing HeLa cells were treated with 4 µM MeHg for the indicated periods. Cells were harvested and the total lysates were subjected to immunoblotting with the indicated antibodies. (b) WT and MerB-expressing HeLa cells were treated with 4 µM MeHg for the indicated periods and then treated with 1% TritonX-100 for 15 min at 4 °C. Solubilized proteins were removed by centrifugation. Soluble and insoluble (●) fractions were subjected to immunoblotting with the indicated antibodies.

**Supplementary Figure S3**

**
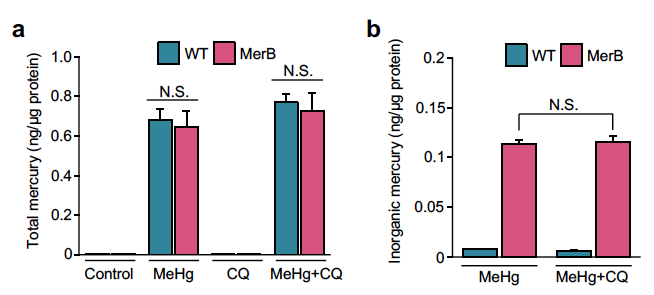
**

(a) WT and MerB-expressing HEK293 cells were treated with 4 µM MeHg for 24 h in the absence or presence of the CQ and the concentration of total mercury in the cells was measured using Mercury Analyzer MA-2. (b) Whole cell lysates were acidified with 200 µL 6 N hydrochloric acid, and then methylmercury was removed by vigorous shaking with toluene in a micro-tube mixer. After the toluene was removed using petroleum ether, each sample was neutralized using 1.71 N sodium hydroxide and used for mercury analysis. Two-group comparisons were performed with Welch Two Sample t-test, N.S., not significant.

**Supplementary Figure S4**

**
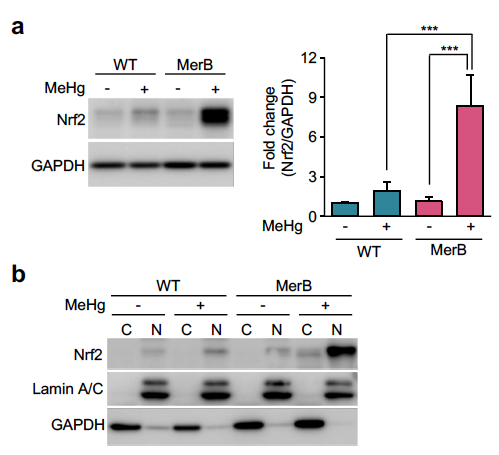
**

(a) WT and MerB-expressing HEK293 cells were treated with 4 µM MeHg for 8 h. Cells were harvested, and the total lysates were subjected to immunoblotting with the indicated antibodies. Immunoblot images (left panel) and intensities (right panels) of WT and MerB-expressing cells were acquired as described in the methods section. (b) WT and MerB-expressing HEK293 cells were treated with 4 µM MeHg for 8 h and lysed with 1% NP-40 and separated cytoplasmic (C) and nucleus (N) fractions as described in the methods section. Both fractions were subjected to immunoblotting with the indicated antibodies.
